# Dissecting the Critical Factors for Thermodynamic Stability of Modular Proteins Using Molecular Modeling Approach

**DOI:** 10.1371/journal.pone.0098243

**Published:** 2014-05-21

**Authors:** Yuno Lee, Joong-jae Lee, Songmi Kim, Sang-Chul Lee, Jieun Han, Woosung Heu, Keunwan Park, Hyun Jung Kim, Hae-Kap Cheong, Dongsup Kim, Hak-Sung Kim, Keun Woo Lee

**Affiliations:** 1 Division of Applied Life Science (BK21 Program), Systems and Synthetic Agrobiotech Center (SSAC), Plant Molecular Biology and Biotechnology Research Center (PMBBRC), Research Institute of Natural Science (RINS), Gyeongsang National University (GNU), Jinju, Korea; 2 Department of Biological Sciences, Korea Advanced Institute of Science and Technology, Daejon, Korea; 3 Department of Bio and Brain Engineering, Korea Advanced Institute of Science and Technology, Daejeon, Korea; 4 Division of Magnetic Resonance Research, Korea Basic Science Institute, Cheongwon, Chungbuk, Korea; University of South Florida College of Medicine, United States of America

## Abstract

Repeat proteins have recently attracted much attention as alternative scaffolds to immunoglobulin antibodies due to their unique structural and biophysical features. In particular, repeat proteins show high stability against temperature and chaotic agents. Despite many studies, structural features for the stability of repeat proteins remain poorly understood. Here we present an interesting result from *in silico* analyses pursuing the factors which affect the stability of repeat proteins. Previously developed repebody structure based on variable lymphocytes receptors (VLRs) which consists of leucine-rich repeat (LRR) modules was used as initial structure for the present study. We constructed extra six repebody structures with varying numbers of repeat modules and those structures were used for molecular dynamics simulations. For the structures, the intramolecular interactions including backbone H-bonds, van der Waals energy, and hydrophobicity were investigated and then the radius of gyration, solvent-accessible surface area, ratio of secondary structure, and hydration free energy were also calculated to find out the relationship between the number of LRR modules and stability of the protein. Our results show that the intramolecular interactions lead to more compact structure and smaller surface area of the repebodies, which are critical for the stability of repeat proteins. The other features were also well compatible with the experimental results. Based on our observations, the repebody-5 was proposed as the best structure from the all repebodies in structure optimization process. The present study successfully demonstrated that our computer-based molecular modeling approach can significantly contribute to the experiment-based protein engineering challenge.

## Introduction

Modular proteins are among the most abundant classes of naturally occurring protein–protein interaction modules [1]. Such proteins are characterized by a linear assembly of consecutive homologous-structural modules comprising a few hundreds to tens of amino acids, showing a modular architecture [2], [3]. Modular proteins have been identified in a variety of functionally related proteins, and their modular architecture has evolved to be suitable for protein-protein interactions, mediating many important biological functions including cell adhesion, signaling process, neural development, bacterial pathogenicity, extracellular matrix assembly, and immune response [4]–[7]. Due to their unique structural and biophysical features, modular proteins have recently attracted much attention as alternative scaffolds to immunoglobulin antibodies [8]. Although immunoglobulin antibodies are widely used in biotechnology and biomedical fields, they have some intrinsic drawbacks. Much effort has been made to develop the alternatives, and a number of diverse protein scaffolds have been reported [9].

**Figure 1:**
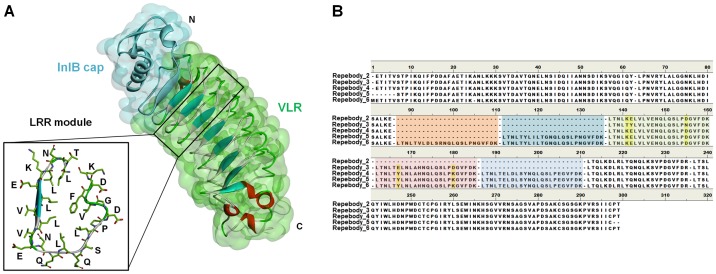
Structure and sequence of repebody. (A) Structural information of repebody which consists of InIB cap (cyan) and VLR (green). LRR module is highlighted in a box and displayed as stick model. (B) Sequence comparison of five different repebodies. The LRR modules are highlighted with different colors; LRR module 1 with orange, 2 with bluish green, 3 with green, 4 with red, with blue. Variable residues are shown as yellow.

We previously developed the repebody scaffold based on variable lymphocyte receptors (VLRs) which are composed of Leucine-rich repeat (LRR) modules (**Fig. 1A**) and involved in adaptive immune response in jawless vertebrates [10], [11]. LRR proteins are abundant in nature, and more than 2,000 proteins have been identified from viruses to eukaryote [12]. The repebody scaffold was shown to have key features for an ideal platform, devoid of the limitations found in immunoglobulin antibodies. In particular, the repebody showed high stability over a wide range of temperature and pH. Because of its intrinsic property and simplicity of the modular architecture, the repebody was revealed to have a potential to be developed as alternative scaffold for the use in the areas of therapeutic proteins, biosensors, and bioseparations.

Here, we present structural analysis for the biophysical property of modular proteins using molecular dynamics (MD) simulations. Previously developed repebody scaffold was used as a model protein. The repebody scaffolds with varying numbers of repeat modules were constructed and subjected to MD simulations to investigate thermodynamic and structural aspects of the proteins in terms of backbone H-bond, energy, radius of gyration (*R*g), solvent-accessible surface area (SASA), secondary structure formation, and hydration free energy. Details are reported herein.

## Results and Discussion

### Construction and model structures of the scaffolds

We constructed the repebodies with varying numbers of consensus designed LRR module composed of 24 residues (LTNLXXLXLXXNQLQSLPXGVFDK) as shown in **Fig. 1B**. The resulting repebodies were designated based on the number of repeat modules: repebody-2 with two modules, repebody-3 with three modules, repebody-4 with four modules, repebody-5 with five modules, and repebody-6 with six modules. Although the five residues in only repebody-3 were observed as variable residues, the residues are located in variable region and exposed part, which made less contribution to the formation of secondary structure. The crystal structure of repebody-5 was used as a template to construct the model structures of other repebodies. To refine the side chains of the model structures, homology modeling was performed.

### Validation of Structural ensembles

**Figure 2:**
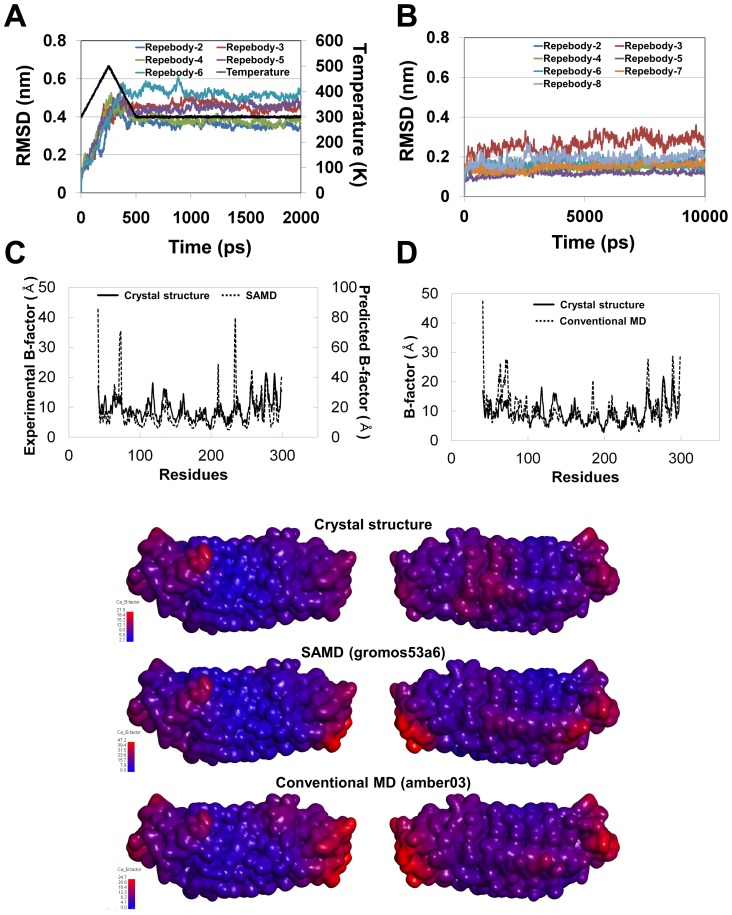
RMSD values of repebodies with different numbers of LRR module under simulated annealing (A) and conventional procedure (B). Comparison of B-factors between crystal and simulated annealing (C) or conventional MD (D) structures. (E) B-factor contour maps on the surface of repebody-5 based on different simulation methods. Predicted B-factor values based on simulated RMSF were obtained by using the conversion formula: B-factor = (8π^2^/3)RMSF^2^ [32].

Our experimental results revealed that the thermodynamic stability of the scaffold increased in proportion to the number of repeat modules. To analyze the relationship between the structural stability and module numbers, we performed simulated annealing molecular dynamics (SAMD) and conventional MD simulations for the repebodies with different module numbers. The simulated annealing procedure was used to overcome computing constraint and local energy barriers and to refine the modeled structure. The conventional MD simulations were also conducted to obtain more stable initial configurations which will be used for calculating free energy of hydration. The root mean square deviations (RMSDs) of Cα atoms were calculated with each initial structure as a reference (**Fig. 2A and B**). In case of SAMD simulation, as the temperature changed from 300 to 500 K linearly during first 250 ps, Cα RMSD values of all systems were dramatically increased to around 0.5 nm and then maintained stably during the rest of simulation time. This result indicates that all the systems were well converged after 1,000 ps and adjusted to heat stress condition. In conventional MD simulation, all the repebodies are stabilized at between approximately 0.1 nm and less than 0.3 nm, which indicates a negligible change in the overall structure.

To check the quality of these ensembles, predicted B-factor value of repebody-5 was calculated based on root mean square fluctuation (RMSF) obtained from these simulation trajectories and compared with crystallographic B-factor (**Fig. 2C and D**). Although the predicted B-factor values of some residues located in loop regions are larger than the experimental values, the predicted values for both SAMD and conventional MD simulations are in good agreement with crystallographic B-factor data. While the value of SAMD was relatively more fluctuated than that of the conventional MD due to the high temperature condition, overall trend of structural fluctuations is similar with each other. To clearly show this trend, we plotted the B-factor contour maps on the surface of repebody-5 based on different simulation methods (**Fig. 2E**). In order to assess the structural stabilities of repebodies, the backbone H-bond, hydrophobicity, energies, radius of gyration (*R*g), solvent-accessible surface areas (SASA), and secondary structure were calculated using the result of SAMD.

### Effect of intra-molecular interactions on thermodynamic stability of proteins

The final snapshot obtained from SAMD simulation was used to measure the number of backbone H-bond of each protein for checking the structural stability (**Table 1**). The highest number of backbone H-bond was observed in repebody-6, whereas the lowest number was found in repebody-2 and repebody-3. To identify the most correlated energy property with the number of modules, the short and long range L-J energies, short and long range Coulomb energies, potential, kinetic and total energies were calculated (**Table 1**). Of these energies, long range coulomb energy in reciprocal space (Coul. Recip. energy) was shown to be the most correlated energy property, displaying the highest linear correlation coefficient (*r* = −0.72) between the module numbers and the energy. In addition, hydrophobicity of buried hydrophobic residues was also calculated to check the thermodynamic stability. Each hydrophobicity value of the proteins with increasing module numbers ranging from 2 to 6 was 197.1, 239.7, 262.7, 273.0, and 316.0, respectively (**Table 1**). The hydrophobicity was shown to be most closely correlated (*r* = 0.92) with thermodynamic stability of the proteins. From these calculations, appropriate intra-molecular properties including backbone H-bond and Coul. Recip. The energy and hydrophobicity were found to represent both structural and thermal stabilities of LRR proteins. These intra-molecular features including hydrophobic interaction are likely to lead to more compact and smaller surface area of the proteins, and this is crucial for the thermodynamic stability of the proteins.

**Table 1:** Correlation between melting temperature *T*_m_ and intramolecular interaction properties including number of backbone H-bond, energies, and hydrophobicity.

	No. of consensus LRR module	No. of total residue	Exp. *T*_m_ (°C)	No. of BackboneH-bond	Coul. Recip. (kJ/mol)	Hydro-phobicity^a^
Repebody-2	1	194	61	42	−80643.7	197.1
Repebody-3	2	218	72	41	−95167.3	239.7
Repebody-4	3	242	82	50	−93305.8	262.7
Repebody-5	4	259	83	59	−98758.6	273.0
Repebody-6	5	291	84	65	−125613.0	316.0
Correlation coefficient (*r*)	0.91	0.90		0.81	−0.72	0.92

*T*_m_, melting temperature; Coul. Recip., long range coulomb energy in reciprocal space; LJ, Lennard-Jones energy.

a Hydrophobicity of buried hydrophobic residues.

### Secondary structure analysis

**Figure 3:**
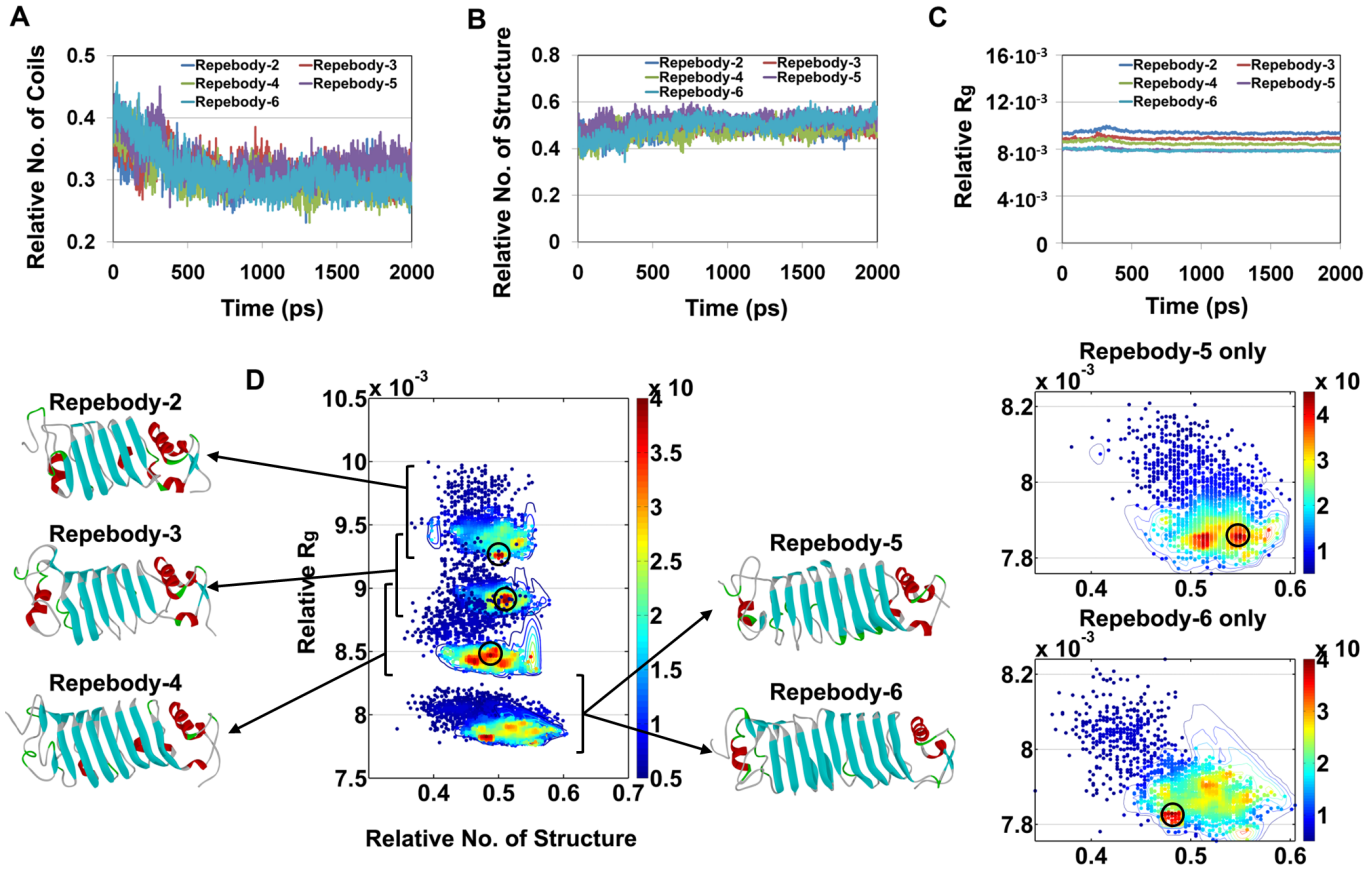
Comparison of structural properties in all the repebody ensembles. Time dependent changes of the secondary structures with relative number of coils (A) and structure (B). (C) Time dependence of the relative gyration (*R*g) for the protein atoms during the simulation time. (D) Contour map of the probability density of relative *R*g as a function of relative number of structure. The most populated conformation for each repebody is highlighted by circle on the map and displayed by ribbon representations. Overlapped region of repebody-5 and 6 in the bottom of the map is separated and displayed into right panel.

To get some insights into the structural stability of repebodies, we analyzed relative number of secondary structures and coils by DSSP approach. The number of coils was calculated and divided by total number of residues (**Fig. 3A**). **Fig. 3B** also shows the ratio of secondary structures among α-helix, β-sheet, β-bridge, and turn with respect to simulation time. Repebody-6 was shown to display the highest ratio (0.529) compared to other repebodies (**Table 2**), whereas the lowest value (0.494) was observed in repebody-2. The composition of secondary structure (*r* = 0.6) for each repebody increased, but the relative number of coils decreased with the increasing number of modules. This result strongly implies that structural stability of the proteins is closely linked with the portion of secondary structures.

**Table 2:** Correlation between melting temperature *T*_m_ and structure parameters including secondary structure, *R*g, density, and SASA.

	No. of consensus LRR module	No. of total residue	Exp. *T*_m_ (°C)	Ratio of secondary structure^a^	Relative *R*g (nm)^a^	Density (g/l)	Ratio of SASA (nm^2^)^a^
Repebody-2	1	194	61	0.494	9.4·10^−3^	1029.67	0.584
Repebody-3	2	218	72	0.496	8.9·10^−3^	1031.48	0.593
Repebody-4	3	242	82	0.490	8.4·10^−3^	1037.46	0.560
Repebody-5	4	259	83	0.527	7.9·10^−3^	1040.39	0.536
Repebody-6	5	291	84	0.529	7.9·10^−3^	1029.68	0.572
Correlation coefficient (*r*)	0.91	0.90		0.60 (0.93)^b^	−0.96	0.55 (0.94)^c^	−0.66 (−0.77)^c^

*T*_m_, Melting temperature; *R*g, Radius of gyration; SASA, solvent-accessible surface area, is in nm^2^ and was calculated using g_sas module in GROMACS.

a Average values during the last 500 ps;

b Value for four data without repebody-4;

c Value for four data without repebody-6.

### Compactness and surface area analysis

To assess the intramolecular compactness of the repebodies, we calculated the relative radius of gyration (*R*g) during the course of the simulation (**Fig. 3C**). Average relative *R*g value over the last 500 ps was shown to represent the clear view for the compactness. Comparison of *R*g values revealed that repebody-6 has the highest compactness, whereas repebody-2 shows the lowest level (**Table 2**). The relative *R*g (*r* = −0.96) values gradually decreased as the number of modules increased. This result indicates that repebodies tend to become more compact as the module number increases. Although the relative *R*g decreased with an increase of the module numbers, the *R*g value of repebody-6 was similar to that of repebody-5. In order to find out which repebody has more intramolecular compactness rather than more sphere-like shape, the density value, which is independent of the protein shape and size, was used to support the relative *R*g (Figure S1). Average value of density for repebodies was indicated that the intramolecular compactness of repebody is increasing in a similar way to the relative *R*g (Table 2). In fact, we have tried other normalized descriptors of compactness such as normalized radius of gyration (Rg/Rg^*^) and coefficient of compactness (S_ASA_/S^*^_ASA_)[13]. But, those descriptors representing the lower value the more sphere-like shape seems inadequate way to explain intramolecular compactness of these repebodies, which are elongated conformation rather than sphere-like one. Next, we investigated the probability distribution of the relative number of structure and relative *R*g to identify the relationship between these properties (**Fig. 3D**). Despite the structural difference between repebody-5 and 6, composition of secondary structure and compactness of overall structure are observed in repebody-5, and these structural features correspond to the most populated structure in repebody-6. This result suggests that repebody-5 is the most optimized structure with higher probability of forming secondary structure and compact conformation compared with other repebodies.

Next, we analyzed the hydrophobic, hydrophilic, total, and SASA to understand the effect of packing. In general, the measurement of SASA is used to assess the protein stability [14]. Lower SASA indicates higher thermodynamic stability of protein. The relative SASA values (*r* = −0.66) of LRR module structures were measured (**Table 2**) to compare the thermodynamic stability of the proteins. Since the SASA of repebody-6 was relatively higher than expected, the correlation was relatively lower than other parameters. However, the correlation was estimated to be −0.77 except repebody-6. As expected from the above analyses, repebody-5 was shown to have most optimized structure, displaying lowest relative SASA value. Furthermore, the lowest energy (203.26) was found in relative short range L-J energy analysis (**Table 3**), which indicates that repebody-5 has lower van der Waals interaction energy compared to the other repebodies. Of the intramolecular interactions, the hydrophobicity showed the highest correlation feature (*r* = −0.95). In addition, hydrophobic core residues in repeat modules tend to pack together like jigsaw pieces, optimizing van der Waals interactions. These interactions are likely to result in more compact structure and smaller surface area, leading to high thermodynamic stability of proteins. These results provide crucial insight into why modular proteins with high number of modules are more stable.

**Table 3:** Relative short rang LJ energy values for selection of optimized VLR module protein.

	No. of consensus LRR module	No. of total residue	Relative short range LJ
Repebody-2	1	194	258.31
Repebody-3	2	218	290.91
Repebody-4	3	242	214.02
Repebody-5	4	259	203.26
Repebody-6	5	291	283.45

### Stability against chaotic agents and solvent accessibility

We tested the stability of repebodies in the presence of different concentrations of urea. CD analysis revealed that the stability against urea also increases with the increasing number of modules (**Table 4**). In order to better understand this tendency, we determined the total solvent-accessible surface area (SASA) of repebodies. Previous studies have shown that unfolding of proteins occurs due to direct interaction of proteins with urea [15], [16]. This interaction can be reduced through an enhanced solvent accessibility for water rather than the urea [17]. Our SASA analysis also showed that total SASA became higher as the module numbers increased. The C_m_ (M) and SASA values increased with an increase in the module number. We found a positive correlation (*r* = 0.82) between the number of C_m_ (M) values and SASA (**Table 4**). This result indicates that higher number of repeat modules leads to higher stability of the proteins against chaotic agents.

**Table 4:** Correlation between concentration of urea C_m_ and SASA.

	Exp. C_m_ (M)	Calc. SASA (nm^2^)
Repebody-2	3.1	294.613
Repebody-3	5.1	329.064
Repebody-4	6.3	340.948
Repebody-5	7.0	345.345
Repebody-6	7.5	432.342
Correlation coefficient (*r*)		0.82

C_m_, concentration of urea.

### Optimal module numbers based on hydration free energy

Our study revealed that repebody-4 has a similar melting temperature to repebody-5 and 6, suggesting that higher number of repeat modules is not directly associated with enhanced thermodynamic stability. To identify optimal number of repeat modules, we further constructed repebody-7 and 8 structures, and calculated their free energies of hydration. Water is known to play a key role in the stabilization of proteins and optimization of their functions [18], [19]. Hence, we reasoned that free energy of hydration *ΔG_hyd_* might reflect the stability and function of a protein in aqueous solution at constant temperature and pressure. We calculated the *ΔG_hyd_* values for all repebodies to identify the optimal number of repeat modules. Our thermodynamic integration calculations showed the lowest values of *ΔG_hyd_* (−336.911 and −335.044) for repebody-4 and 5, respectively (**Table 5**), suggesting that the optimal number of repeat module is three or four.

**Table 5:** Comparison of free energies of hydration for all seven repebodies.

System	Van der Waals	Columbic	Total (kJ/mol)	Relative *ÄG_hyd_*
**Repebody-2**	−2979.20±40.00	−62330.20±59.22	−65309.4±99.22	−336.646
**Repebody-3**	−3354.78±16.12	−69093.44±54.00	−72448.22±70.12	−332.331
**Repebody-4**	−3786.47±11.32	−77746.01±50.61	−81532.48±61.93	−336.911
**Repebody-5**	−4030.70±15.30	−82745.75±53.32	−86776.45±68.64	−335.044
**Repebody-6**	−4334.05±67.71	−92479.39±95.79	−96813.44±163.5	−332.692
**Repebody-7**	−4704.79±39.08	−99825.86±75.24	−104530.65±114.32	−331.843
**Repebody-8**	−4985.07±18.52	−107255.02±98.74	−112240.09±117.26	−331.092

Relative *ΔG_hyd_*, Relative free energy of hydration, is total energy divided by number of residues.

### Conclusions

We have demonstrated that thermodynamic stability of the modular protein composed of LRR modules is significantly affected by the number of constituting modules. Our molecular modeling study provides the critical physicochemical features affecting the thermodynamic stability of the modular protein. Our study showed that the backbone H-bond, long range coulomb, van der Waals energies, and hydrophobicity make significant contribution to the stability of the modular protein. The *R*g, SASA, and ratio of secondary structure were also consistent with the experimental measurements for the thermodynamic stability of the protein. Our results revealed that an increase in the module numbers results in more compact structure and smaller surface area of the repebodies, leading to high thermodynamic stability of the proteins due to increased intramolecular interactions. The stability of the protein against chaotic reagents has also revealed a positive correlation between the number of repeat modules and the stability. Our results will provide crucial insight into designing modular proteins with high thermodynamic stability for practical applications as alternative scaffolds.

## Materials and Methods

### Gene synthesis, expression, and protein purification

The scaffolds with different numbers of LRR modules were constructed by overlapping PCR using the parental Repebody scaffold as a template sequence, and their nucleotide sequences are listed in Table S1. The genes were cloned into pET21a vector (Invitrogen) between the NdeI and XhoI restriction enzyme sites with a hexa-histidine tag at the C-terminal for affinity purification. The vectors including the respective genes were transformed into Origami-BTM *E. coli* cells (Merck Biosciences) to enhance disulfide bond formation. Cells were grown in LB media at 37°C until the absorbance at 600 nm reached 0.5. Isopropyl β-D-1-thiogalactopyranoside (IPTG) was then added at a final concentration of 0.5 mM for induction. Cells were further incubated at 18°C for 20 hrs, harvested by centrifugation at 4,000 g, and were suspended in a lysis buffer (pH 8.0) containing 50 mM NaH2PO4, 300 mM NaCl and 10 mM immidazole. Cells were disrupted by sonication, followed by centrifugation at 12,000 rpm for 15 min, and supernatants were collected for protein purification. The cleared lysates were purified by affinity chromatography using Ni-NTA Superflow (Qiagen). A protein solution was applied to the resin-packed column, followed by washing with a solution containing 20 mM immidazole until no protein was detected by Bradford assay. Bound proteins were eluted with elution buffer containing 250 mM immidazole, and fractions were collected. For circular dichroism analysis, proteins were further purified over a desalting column (Pierce) and PBS buffer (pH 7.4) to remove high salts in the elution buffer.

### Circular dichroism analysis

Molar ellipticities of proteins were measured from 190 nm to 280 nm at 25°C using circular dichroism (Jasco J-815) to check their secondary structures. Melting temperatures of proteins were determined by measuring molar ellipticity at 222 nm with a gradual increase in temperature from 25°C to 90°C. The effect of pH on proteins was also investigated using circular dichroism. Proteins were incubated for 24 hr in buffers with different pH values and molar ellipticities at 222 nm were measured from 65°C to 90°C. The buffers used included: 0.3 M NaCl, 5 mM glycine for pH 3.0, citrate buffer (5 mM) for pH 4.0, and 10 mM sodium phosphate for pH 5.0 to 12.0. The midpoint of transition was designated as the melting temperature of the protein.

### Model structures of repebodies

The model structures of repebodies with different LRR module numbers were obtained by Discovery Studio (DS) 2.5 using the structure of the repebody with five LRR modules (Repebody-5: PDB ID code 3RFS) as a template. Homology modeling was performed to refine these modified structures using the Build Homology Models protocols in DS 2.5.

### Simulated annealing molecular dynamics simulations

One crystal structure and four modeled structures were subjected to simulated annealing molecular dynamics (SAMD) simulations during 2 ns [20]. The five MD simulations were performed with GROMACS program (version 4.5.1; http://www.gromacs.org/) [21], [22] using GROMOS96 force field [23]. Those structures were immersed in an orthorhombic water box. MD simulations were carried out in explicit solvent using a simple point charge (SPC) model, and Na^+^ counter ions were added to neutralize the net charge of the system. The entire systems are made up of approximately 26,500 to 43,000 atoms with about 8,400 to 13,600 water molecules. The energy minimization of the initial systems was carried out using the conjugate gradients algorithm until a tolerance of 200 kJ/mol. The systems were subjected to position-restrained MD simulation at 300 K and normal pressure (1 bar) for 100 ps. The production run was performed by increasing the temperature from 300 to 500 K symmetrically within 250 ps, and by decreasing the temperature to 300 K linearly during 250 ps and then stabilizing at 300 K for 1500 ps. The five production runs were carried out under periodic boundary conditions with NPT ensemble and V-rescale thermostat [24]. The LINCS algorithm was used to constrain all bond-lengths [25] and finally the particle mesh Ewald (PME) method [26] was also used to manage the long range electrostatics.

### Molecular dynamics simulations with thermodynamic integration calculations

Prior to thermodynamic integration (TI) calculations, 10 ns conventional MD simulations of the five structures mentioned in above and additional two repebodies (repebody-7 and 8) were conducted with AMBER03 force field [27], [28] to obtain more stable initial configurations. The TI calculations were used to estimate the relative free energy of hydration for the all repebodies. The difference of hydration free energy, *ΔG_hyd_*, between the solvent-decoupled (λ = 0) and fully solvent-coupled (λ = 1) states is obtained using the TI formula [29], [30]: 
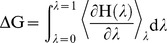



Totally, 42 λ points were used for introducing van der Waals interactions first and charging later with λ spacing value of 0.05. At each value of λ, the repebody systems were energy-minimized using steepest descent and L-BFGS methods and then equilibrated for 100 ps under each different ensembles: isochoric-isothermal (NVT) and isothermal-isobaric (NPT). Production runs were performed for 1 ns under an NPT ensemble.

### Analysis of structural stability

The root mean square deviation (RMSD), radius of gyration (*R*g), and energies were calculated using GROMACS analysis tools in order to assess structural stabilities of the repebodies. The *R*g value of each protein was divided by total number of residues to evaluate their relative compactness. 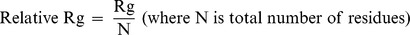



The *g_energy* module in the analysis tool was used to calculate the short and long range Lennard-Jones (LJ) energies, short and long range coulomb energies, potential, kinetic, and total energies. The number of backbone H-bond and hydrophobicity in each final snapshot were also calculated using DS 2.5. Secondary structure analysis was carried out using the DSSP (Define Secondary Structure of Proteins) algorithm which is characterizing the time-dependent secondary structure fluctuation [31]. Hydrophobic, hydrophilic, total, solvent-accessible surface areas, and density of protein were calculated by the *g_sas* module in the GROMACS analysis package.

## Supporting Information

Figure S1 Time dependence of density (mass/volume) for the repebodies during the simulation time.
(TIF)

Table S1 Nucleotide sequences of constructed repebodies.
(DOCX)
