# Supplementary figures and images for: Dissecting the Critical Factors for Thermodynamic Stability of Modular Proteins Using Molecular Modeling Approach
Source: PLoS One. 2014 May 21;9(5):e98243. doi: 10.1371/journal.pone.0098243 (PMC4029881; doi:10.1371/journal.pone.0098243)

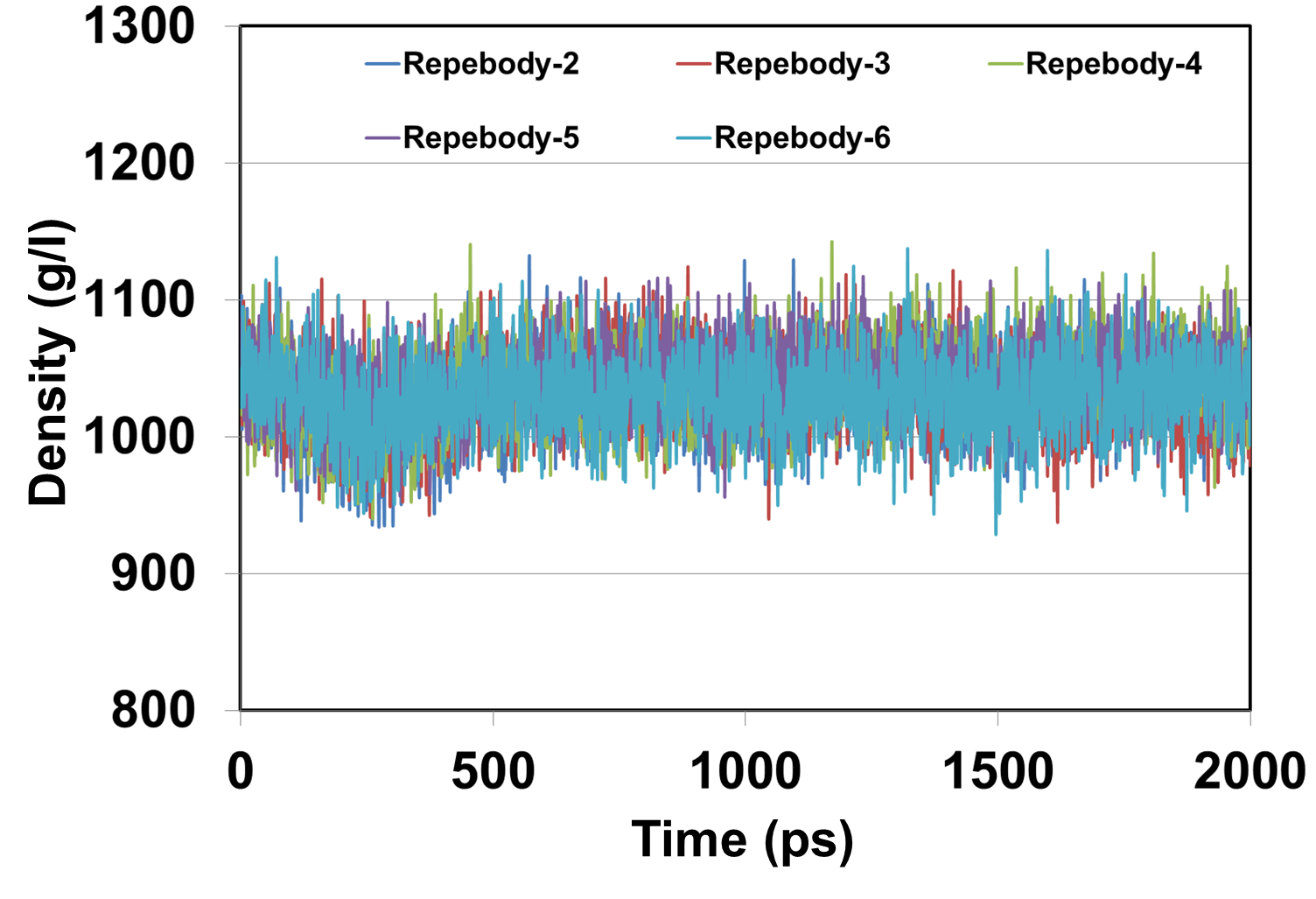

Supplement: Figure S1 — Time dependence of density (mass/volume) for the repebodies during the simulation time. (TIF) [file pone.0098243.s001.tif]
