# Supplementary material for: Dissecting the Critical Factors for Thermodynamic Stability of Modular Proteins Using Molecular Modeling Approach
Source: PLoS One. 2014 May 21;9(5):e98243. doi: 10.1371/journal.pone.0098243 (PMC4029881; doi:10.1371/journal.pone.0098243)
Supplement: Table S1 — Nucleotide sequences of constructed repebodies. (DOCX) [file pone.0098243.s002.docx]

Table S1. Nucleotide sequence of five different repebodies

| Repebody-2 (582 bp) |
| --- |
| gaaaccatca ccgtttctac cccgatcaaa cagatcttcc cggacgacgc tttcgctgaa 60  accatcaaag ctaacctgaa aaaaaaatct gttaccgacg ctgttaccca gaacgaactg 120  aactctatcg accagatcat cgctaacaac tctgacatca aatctgttca gggtatccag 180  tacctgccga acgttcgtta cctggctctg ggtggtaaca aactgcacga catctctgct 240  ctgaaagaac tgaccaacct gaaagaactg gttctggttg aaaaccagct gcagtctctg 300  ccggacggtg ttttcgacaa actgacccag ctgaaagacc tgcgtctgta ccagaaccag 360  ctgaaatctg ttccggacgg tgttttcgac cgtctgacct ctctgcagta catctggctg 420  cacgacaacc cgtgggactg cacctgcccg ggtatccgtt acctgtctga atggatcaac 480  aaacactctg gtgttgttcg taactctgct ggttctgttg ctccggactc tgctaaatgc 540  tctggttctg gtaaaccggt tcgttctatc atctgcccga cc |
| Repebody-3 (654 bp) |
| gaaaccatca ccgtttctac cccgatcaaa cagatcttcc cggacgacgc tttcgctgaa 60  accatcaaag ctaacctgaa aaaaaaatct gttaccgacg ctgttaccca gaacgaactg 120  aactctatcg accagatcat cgctaacaac tctgacatca aatctgttca gggtatccag 180  tacctgccga acgttcgtta cctggctctg ggtggtaaca aactgcacga catctctgct 240  ctgaaagaac tgaccaacct gacctacctg aacctggctc acaaccagct gcagtctctg 300  ccgaaaggtg ttttcgacaa actgaccaac ctgaccgaac tggacctgtc ttacaaccag 360  ctgcagtctc tgccggaagg tgttttcgac aaactgaccc agctgaaaga cctgcgtctg 420  taccagaacc agctgaaatc tgttccggac ggtgttttcg accgtctgac ctctctgcag 480  tacatctggc tgcacgacaa cccgtgggac tgcacctgcc cgggtatccg ttacctgtct 540  gaatggatca acaaacactc tggtgttgtt cgtaactctg ctggttctgt tgctccggac 600  tctgctaaat gctctggttc tggtaaaccg gttcgttcta tcatctgccc gacc |
| Repebody-4 (726 bp) |
| gaaaccatca ccgtttctac cccgatcaaa cagatcttcc cggacgacgc tttcgctgaa 60  accatcaaag ctaacctgaa aaaaaaatct gttaccgacg ctgttaccca gaacgaactg 120  aactctatcg accagatcat cgctaacaac tctgacatca aatctgttca gggtatccag 180  tacctgccga acgttcgtta cctggctctg ggtggtaaca aactgcacga catctctgct 240  ctgaaagaac tgaccaacct gaaagaactg gttctggttg aaaaccagct gcagtctctg 300  ccggacggtg ttttcgacaa actgaccaac ctgacctacc tgaacctggc tcacaaccag 360  ctgcagtctc tgccgaaagg tgttttcgac aaactgacca acctgaccga actggacctg 420  tcttacaacc agctgcagtc tctgccggaa ggtgttttcg acaaactgac ccagctgaaa 480  gacctgcgtc tgtaccagaa ccagctgaaa tctgttccgg acggtgtttt cgaccgtctg 540  acctctctgc agtacatctg gctgcacgac aacccgtggg actgcacctg cccgggtatc 600  cgttacctgt ctgaatggat caacaaacac tctggtgttg ttcgtaactc tgctggttct 660  gttgctccgg actctgctaa atgctctggt tctggtaaac cggttcgttc tatcatctgc 720  ccgacc |
| Repebody-5 (798 bp) |
| gaaaccatca ccgtttctac cccgatcaaa cagatcttcc cggacgacgc tttcgctgaa 60  accatcaaag ctaacctgaa aaaaaaatct gttaccgacg ctgttaccca gaacgaactg 120  aactctatcg accagatcat cgctaacaac tctgacatca aatctgttca gggtatccag 180  tacctgccga acgttcgtta cctggctctg ggtggtaaca aactgcacga catctctgct 240  ctgaaagaac tgaccaacct gacctacctg atcctgaccg gtaaccagct gcagtctctg 300  ccgaacggtg ttttcgacaa actgaccaac ctgaaagaac tggttctggt tgaaaaccag 360  ctgcagtctc tgccggacgg tgttttcgac aaactgacca acctgaccta cctgaacctg 420  gctcacaacc agctgcagtc tctgccgaaa ggtgttttcg acaaactgac caacctgacc 480  gaactggacc tgtcttacaa ccagctgcag tctctgccgg aaggtgtttt cgacaaactg 540  acccagctga aagacctgcg tctgtaccag aaccagctga aatctgttcc ggacggtgtt 600  ttcgaccgtc tgacctctct gcagtacatc tggctgcacg acaacccgtg ggactgcacc 660  tgcccgggta tccgttacct gtctgaatgg atcaacaaac actctggtgt tgttcgtaac 720  tctgctggtt ctgttgctcc ggactctgct aaatgctctg gttctggtaa accggttcgt 780  tctatcatct gcccgacc |
| Repebody-6 (870 bp) |
| gaaaccatca ccgtttctac cccgatcaaa cagatcttcc cggacgacgc tttcgctgaa 60  accatcaaag ctaacctgaa aaaaaaatct gttaccgacg ctgttaccca gaacgaactg 120  aactctatcg accagatcat cgctaacaac tctgacatca aatctgttca gggtatccag 180  tacctgccga acgttcgtta cctggctctg ggtggtaaca aactgcacga catctctgct 240  ctgaaagaac tgaccaacct gaccgttctg gacctgtctc gtaaccagct gcagtctctg 300  ccgaacggtg ttttcgacaa actgaccaac ctgacctacc tgatcctgac cggtaaccag 360  ctgcagtctc tgccgaacgg tgttttcgac aaactgacca acctgaaaga actggttctg 420  gttgaaaacc agctgcagtc tctgccggac ggtgttttcg acaaactgac caacctgacc 480  tacctgaacc tggctcacaa ccagctgcag tctctgccga aaggtgtttt cgacaaactg 540  accaacctga ccgaactgga cctgtcttac aaccagctgc agtctctgcc ggaaggtgtt 600  ttcgacaaac tgacccagct gaaagacctg cgtctgtacc agaaccagct gaaatctgtt 660  ccggacggtg ttttcgaccg tctgacctct ctgcagtaca tctggctgca cgacaacccg 720  tgggactgca cctgcccggg tatccgttac ctgtctgaat ggatcaacaa acactctggt 780  gttgttcgta actctgctgg ttctgttgct ccggactctg ctaaatgctc tggttctggt 840  aaaccggttc gttctatcat ctgcccgacc |
